# Supplementary material for: APOL1 G1 and G2 risk alleles modulate severity of diet-induced obesity in a transgenic mouse model
Source: bioRxiv. 2025 Dec 25:2025.12.23.696293. Preprint. [Version 1] doi: 10.64898/2025.12.23.696293 (PMC12767306; doi:10.64898/2025.12.23.696293)
Supplement: 1 [file NIHPP2025.12.23.696293V1-supplement-1.pdf]

bioRxiv preprint doi: https://doi.org/10.64898/2025.12.23.696293; this version posted December 25, 2025. The copyright holder for this preprint (which was not certified by peer review) is the author/funder, who has granted bioRxiv a license to display the preprint in perpetuity. It is made available under aCC-BY 4.0 International license.

Supporting Information

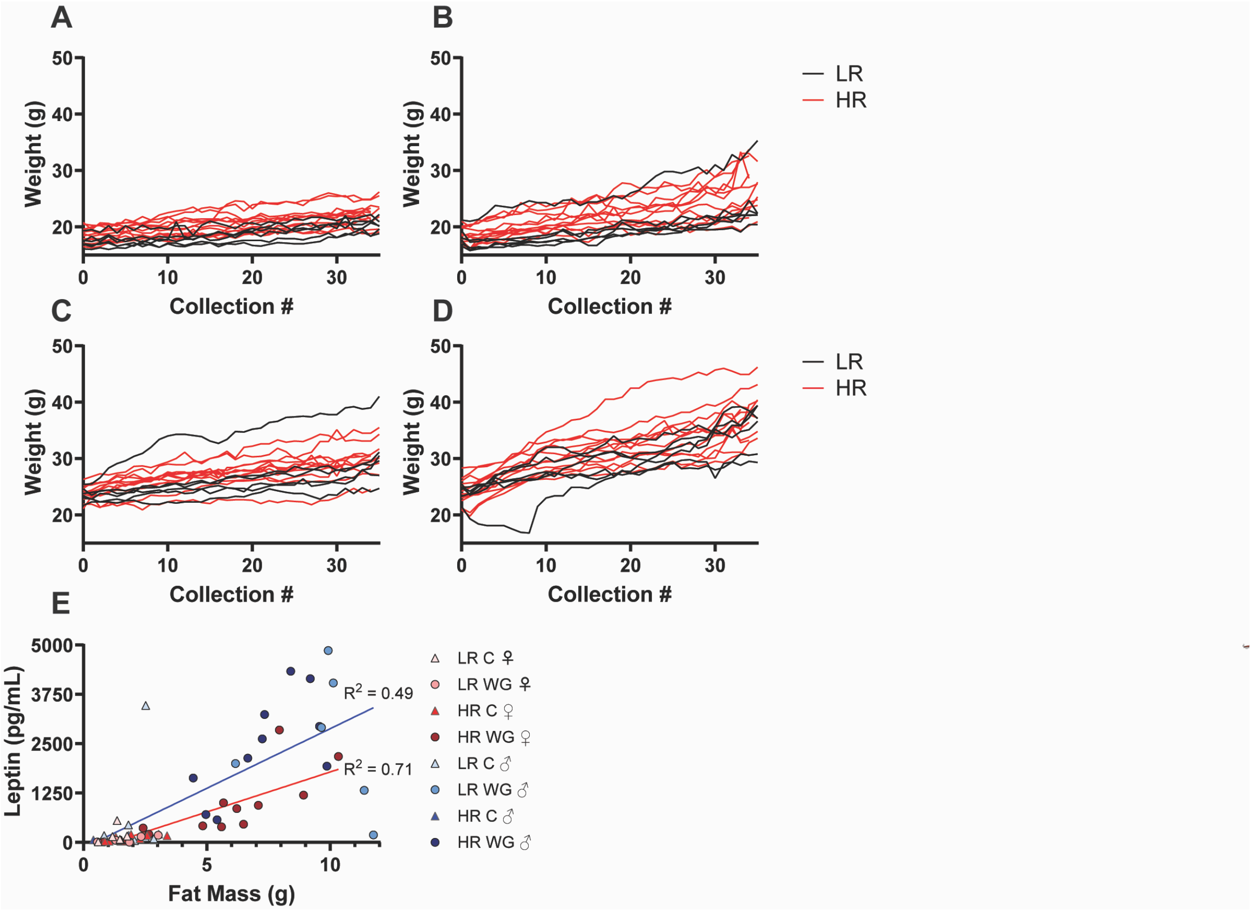

Supplemental Figure S1. Individual gross weight-over-time of 16wk experiment.

A) C Females **B)** WG Females **C)** C Males **D)** WG Males. Collection # increments per instance of being weighed. **E)** Correlation between fat mass and fasted serum leptin level.

C, control; WG, weight gain.

bioRxiv preprint doi: https://doi.org/10.64898/2025.12.23.696293; this version posted December 25, 2025. The copyright holder for this preprint (which was not certified by peer review) is the author/funder, who has granted bioRxiv a license to display the preprint in perpetuity. It is made available under aCC-BY 4.0 International license.

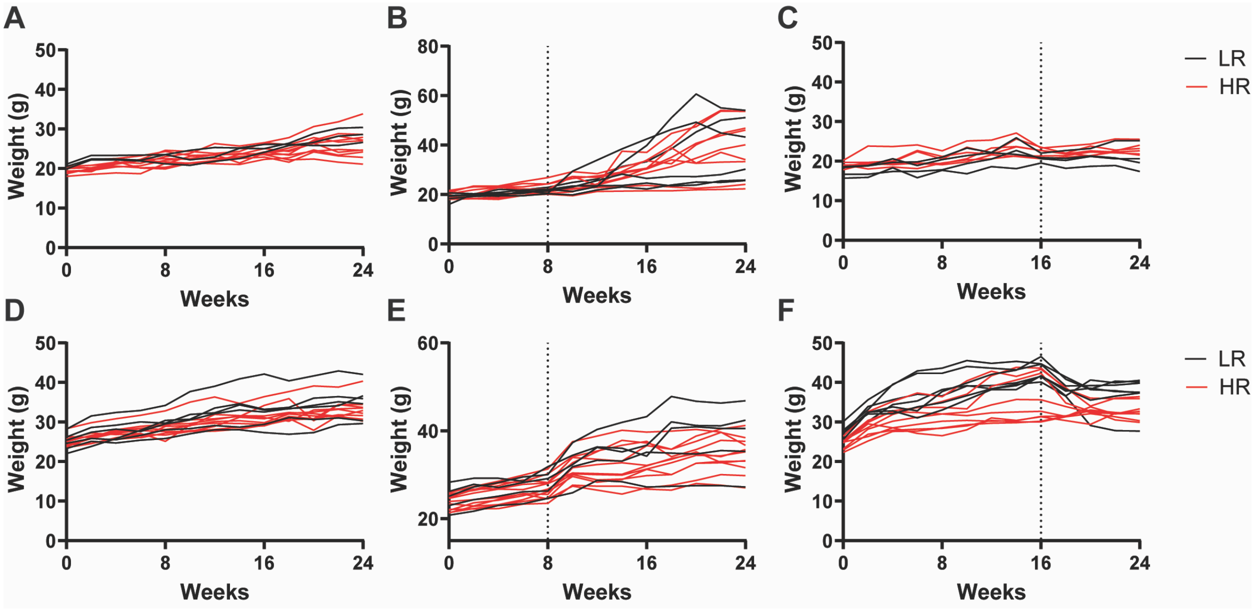

Supplemental Figure S2. Individual gross weight-over-time plots of 24wk experiment.

A) C Females **B)** WG Females **C)** WL Females **D)** C Males **E)** WG males **F)** WL males.

C, control; WG, weight gain; WL, weight loss.

bioRxiv preprint doi: https://doi.org/10.64898/2025.12.23.696293; this version posted December 25, 2025. The copyright holder for this preprint (which was not certified by peer review) is the author/funder, who has granted bioRxiv a license to display the preprint in perpetuity. It is made available under aCC-BY 4.0 International license.

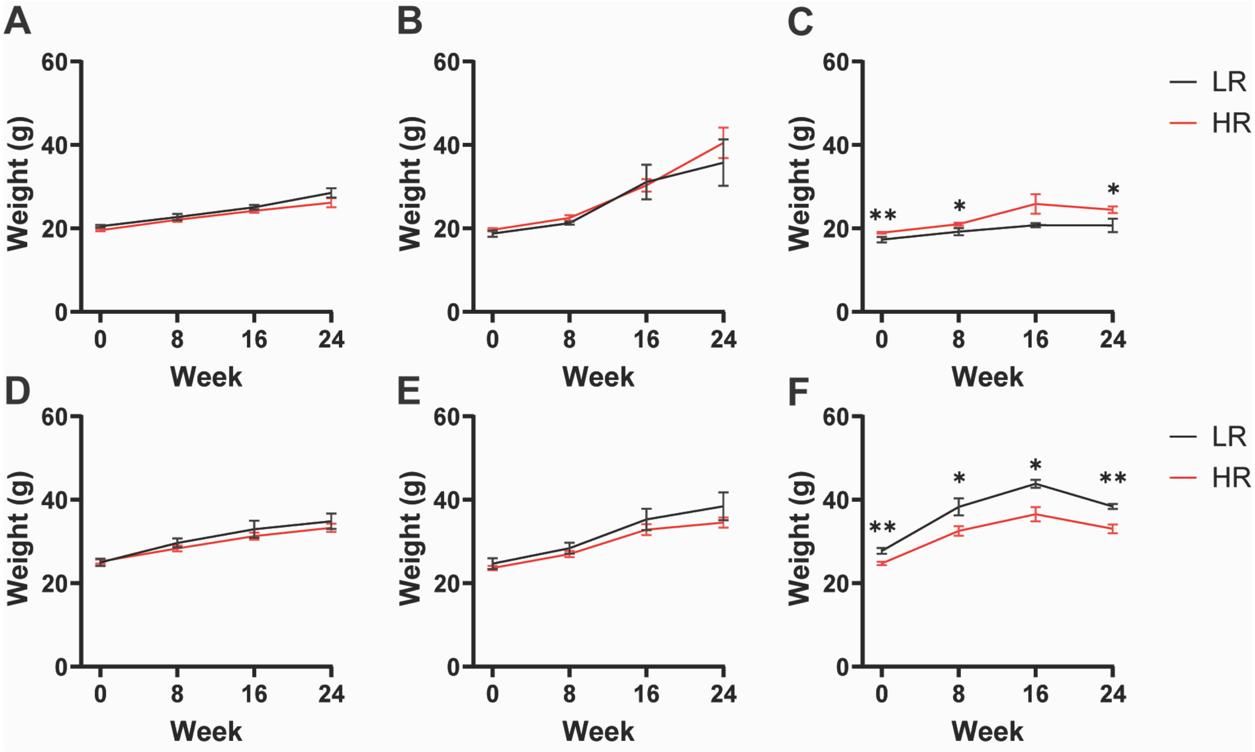

Supplemental Figure S3. Gross weight at 8, 16, and 24 weeks of 24wk experiment.

A) C Females **B)** WG Females **C)** WL Females **D)** C Males **E)** WG males **F)** WL males.

C, control; WG, weight gain; WL, weight loss.

bioRxiv preprint doi: https://doi.org/10.64898/2025.12.23.696293; this version posted December 25, 2025. The copyright holder for this preprint (which was not certified by peer review) is the author/funder, who has granted bioRxiv a license to display the preprint in perpetuity. It is made available under aCC-BY 4.0 International license.

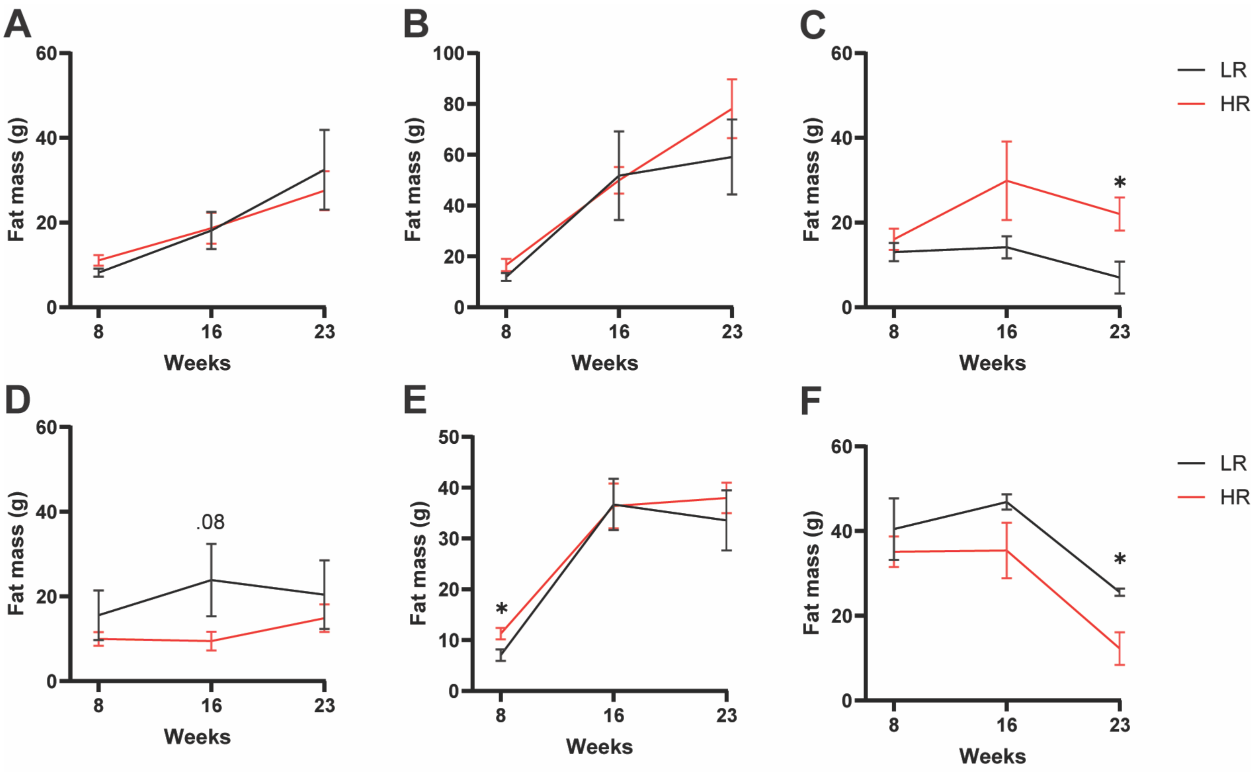

Supplemental Figure S4. Fat mass at 8, 16, and 24 weeks of 24wk experiment.

A) C Females **B)** WG Females **C)** WL Females **D)** C Males **E)** WG males **F)** WL males.

C, control; WG, weight gain; WL, weight loss.

bioRxiv preprint doi: https://doi.org/10.64898/2025.12.23.696293; this version posted December 25, 2025. The copyright holder for this preprint (which was not certified by peer review) is the author/funder, who has granted bioRxiv a license to display the preprint in perpetuity. It is made available under aCC-BY 4.0 International license.

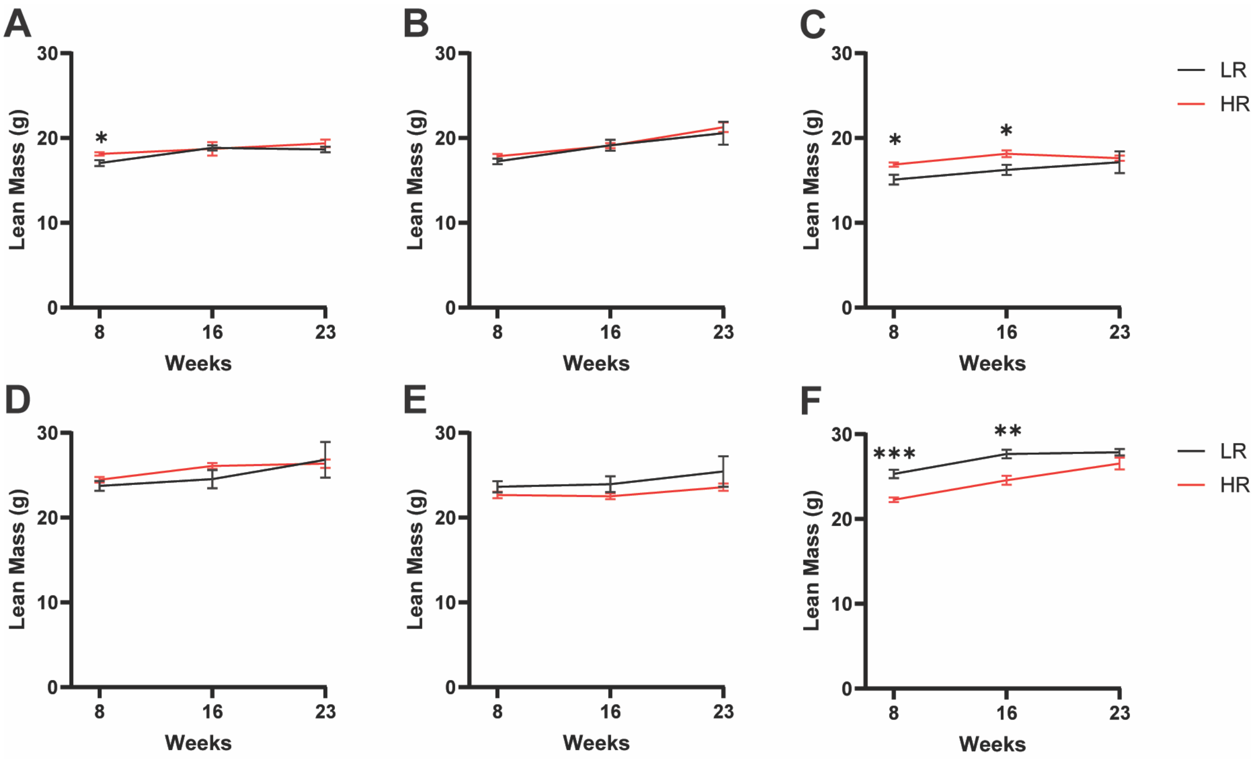

Supplemental Figure S5. Lean mass at 8, 16, and 24 weeks of 24wk experiment.

A) C Females **B)** WG Females **C)** WL Females **D)** C Males **E)** WG males **F)** WL males.

C, control; WG, weight gain; WL, weight loss.

bioRxiv preprint doi: https://doi.org/10.64898/2025.12.23.696293; this version posted December 25, 2025. The copyright holder for this preprint (which was not certified by peer review) is the author/funder, who has granted bioRxiv a license to display the preprint in perpetuity. It is made available under aCC-BY 4.0 International license.

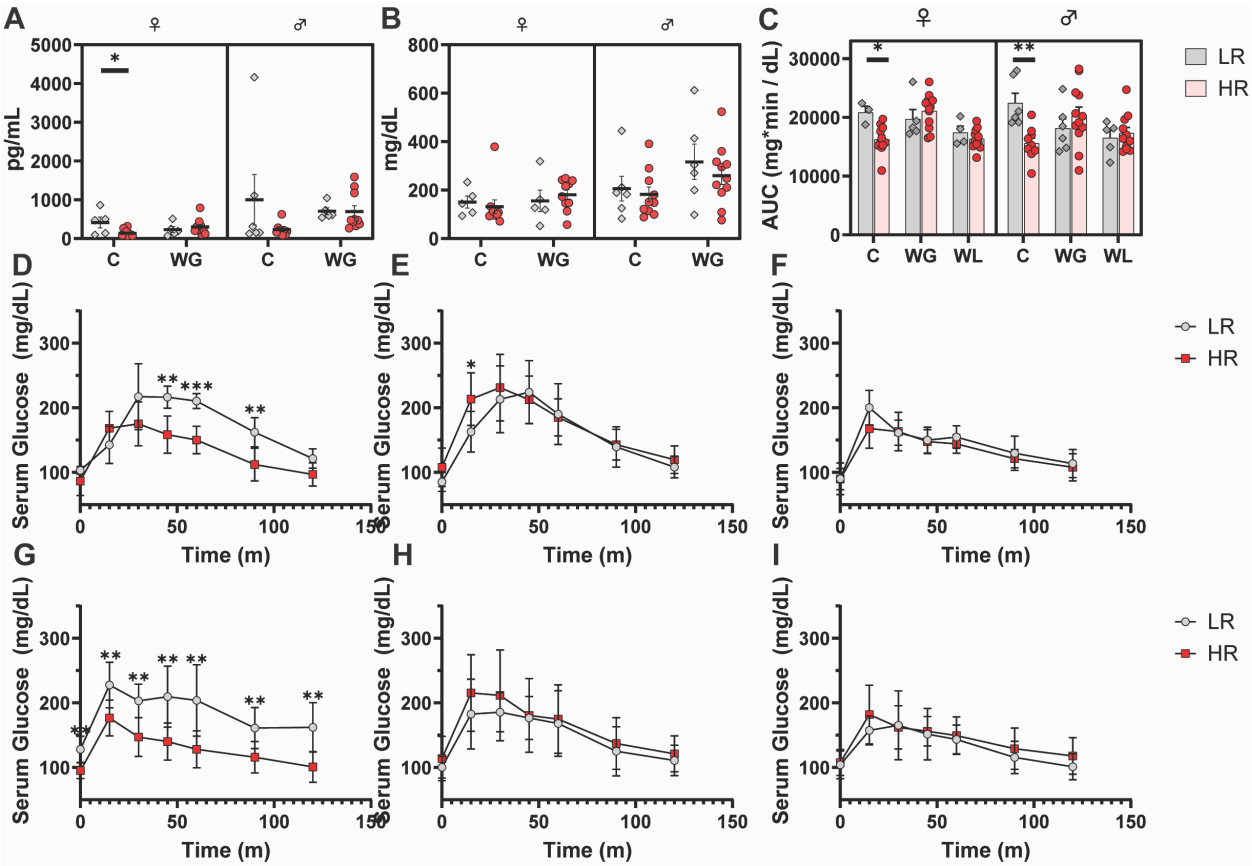

Supplemental Figure S6. oGTT plots.

A) Fasted serum insulin concentration of 16wk experiment. **B)** Fasted serum glucose concentration of 16wk experiment. **C)** Area-under-the-curve of 24wk experiment oGTTs. oGTT curves of 24wk **D)** C Females **E)** WG Females **F)** WL Females **G)** C Males **H)** WG males **I)** WL males.

oGTT, oral glucose tolerance test; C, control; WG, weight gain; WL, weight loss.

bioRxiv preprint doi: https://doi.org/10.64898/2025.12.23.696293; this version posted December 25, 2025. The copyright holder for this preprint (which was not certified by peer review) is the author/funder, who has granted bioRxiv a license to display the preprint in perpetuity. It is made available under aCC-BY 4.0 International license.

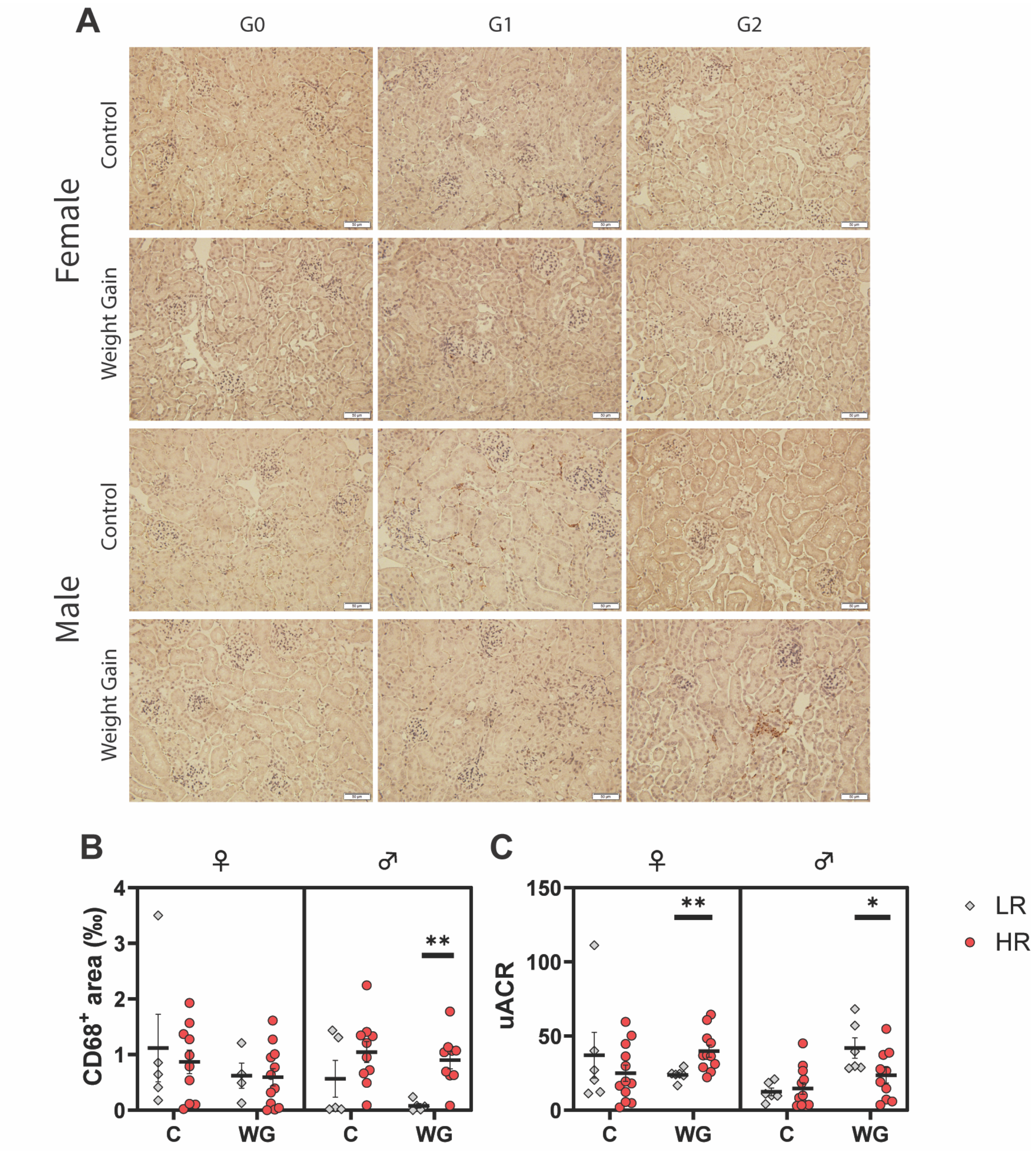

Supplemental Figure S7. Kidney Injury

A) Representative IHC images of kidney bisections stained for CD68. Scale bar, 20μM. **B)** Quantification of CD68-stained kidney bisections collected from short-term (16-week) experiment. **C)** uACR of 16wk experiment.

IHC, immunohistochemistry; uACR, urinary albumin:creatinine ratio.
